# Supplementary material for: Asynchronous magnetic resonance elastography: Shear wave speed reconstruction using noise correlation of incoherent waves
Source: Magn Reson Med. 2022 Oct 27;89(3):990–1001. doi: 10.1002/mrm.29502 (PMC9792433; doi:10.1002/mrm.29502)
Supplement: Supplementary file 1 — DATA S1 MATLAB code used to generate the numerical simulations of Figure 1 [file MRM-89-990-s002.zip › k-Wave/helpfiles/overlayPlot.html]

overlayPlot :: Functions (k-Wave)


# overlayPlot

Overlay two images.

## Syntax

```
overlayPlot(bg, fg)
overlayPlot(bg, fg, ...)
overlayPlot(x, y, bg, fg)
overlayPlot(x, y, bg, fg, ...)
```

## Description

`overlayPlot` overlays two 2D images. The background image is displayed using a grayscale map. For a non-zero dynamic range, the foreground image is log compressed (discarding negative values), thresholded to a particular dynamic range, and overlaid using an alpha value of 0.5. If the dynamic range is set to zero, the foreground image is overlaid without additional processing.

Example:

```
bg = rand(128);
fg = peaks(128);
overlayPlot(bg, fg);
```

## Inputs

|  |  |
| --- | --- |
| `x, y` | vectors describing the position of the pixels in the image equivalent to `image`(x, y, c) |
| `bg` | background image |
| `fg` | foreground image |

## Optional Inputs

Optional 'string', value pairs that may be used to modify the default computational settings.

| Input | Valid Settings | Default | Description |
| --- | --- | --- | --- |
| `'ColorBar'` | *(Boolean scalar)* | `false` | Boolean controlling whether a colorbar is displayed. |
| `'ColorBarTitle'` | *(string)* | `'dB'` with log compression `''` otherwise | String defining the title used for the colorbar. |
| `'ColorMap'` | *(string)* | `'jet'` | String defining the colormap used for the overlay. |
| `'LogComp'` | *(Boolean scalar)* | `true` | Boolean controlling whether the forergound image is log compressed before display. |
| `'LogCompRef'` | *(numeric scalar)* | `max(fg(:))` | Reference value used in the log compression, where `fg_compressed = 20 * log10(fg ./ fg_ref)`. |
| `'NumColors'` | *(integer scalar)* | *256* | Number of colors used in the colormaps. |
| `'PlotScale'` | *(numeric two element vector)* | `[-30, 0]` with log compression  `[min(fg(:)), max(fg(:))]` otherwise | Plot scale used to display the foreground image. |

## See Also

`beamPlot`, `voxelPlot`
